# Tracking of device-measured sedentary time, cardiorespiratory fitness, and cardiometabolic risk factors from childhood to young adulthood

**DOI:** 10.1016/j.ajpc.2025.101313

**Published:** 2025-09-22

**Authors:** Anders Husøy, Elin Kolle, Jostein Steene-Johannessen, Lars Bo Andersen, Ulf Ekelund, Sigmund Alfred Anderssen

**Affiliations:** a Department of Sports Medicine, Norwegian School of Sport Sciences N-0806 Oslo, Norway; b Department of Sport, Food and Natural Sciences, Western Norway University of Applied Sciences N-6856 Sogndal, Norway; c Department of Chronic Diseases, Norwegian Institute of Public Health N-0213 Oslo, Norway

**Keywords:** Childhood, Young adulthood, Tracking, Cardiometabolic risk, Sedentary time, Cardiorespiratory fitness

## Abstract

**Background:**

The tracking of clustered cardiometabolic risk from childhood to young adulthood, i.e., whether an unfavourable cardiometabolic risk profile persists from childhood into young adulthood, remain uncertain and has previously not included factors like sedentary time and cardiorespiratory fitness.

**Methods:**

We used longitudinal data from the Physical Activity among Norwegian Children Studies (PANCS), where 731 participants participated at age 9 (2005–2006) and 258 of these again at age 24 (2019–2021). Multiple imputation was performed for all eligible participants at age 24 (*n* = 708). The cardiometabolic risk factors comprised body mass index (BMI), waist circumference, device-measured sedentary time, directly measured peak oxygen uptake (VO_2peak_), systolic blood pressure, LDL-cholesterol, insulin, high-sensitivity CRP, and a clustered risk Z-score. Tracking was analysed using regression models.

**Results:**

Waist circumference and BMI demonstrated strong tracking, whereas moderate tracking was observed for VO_2peak_, LDL-cholesterol, systolic blood pressure, and clustered cardiometabolic risk. Insulin, high-sensitivity CRP, and sedentary time exhibited no tracking. Unfavourable childhood levels of risk factors demonstrating moderate to strong tracking were highly predictive, with those in the least favourable quartile of clustered risk at age 9 almost six times more likely than their peers to be in the least favourable quartile at age 24 years. Tracking was generally similar between sexes, but waist circumference tracked somewhat stronger among females while VO_2peak_ tracked stronger among males.

**Conclusions:**

Several cardiometabolic risk factors demonstrated moderate to strong tracking from childhood to young adulthood, supporting early intervention. However, sedentary behaviour may be less stable and easier to modify compared with more stable cardiometabolic risk factors.

## 1 Introduction

Cardiovascular diseases (CVD) continues to be the leading global cause of mortality, estimated to account for >20 million deaths in 2021 while imposing a substantial burden of disability and excess health costs [1,2]. Cardiometabolic risk factors all contribute to development of the atherogenic process and type 2 diabetes mellitus and tend to cluster together [3,4], thereby increasing the risk for CVD [5,6]. Risk factors include obesity, high blood pressure, high cholesterol levels, high fasting levels of glucose and insulin, sedentary behaviour, and low cardiorespiratory fitness [[7], [8], [9]]. Tracking of cardiometabolic risk could be defined as either the stability of these risk factors over time or the predictability of values later in life from early life measurements [10], with the underlying aim of both definitions to examine whether an unfavourable cardiometabolic risk profile persists over time. It is recognised that the onset of CVD may start already in childhood [11,12], suggesting implementation of early action to prevent further development of those risk factors that demonstrate a high level of tracking.

The transition from childhood to adulthood often involves major life changes, and this period has been identified as key for disease prevention [13]. Previous studies have demonstrated that unfavourable levels of several cardiometabolic risk markers in childhood are likely to persist into adulthood, particularly measures of obesity [10,[14], [15], [16]]. However, it is unclear how device-measured sedentary time and directly measured VO_2peak_ may track over this transitional period. Moreover, it is unclear whether a clustered risk score including sedentary time and VO_2peak_ tracks from childhood to young adulthood and whether tracking differ by sex [14].

We aimed to examine the tracking of several cardiometabolic risk factors individually and as a clustered risk score, including sedentary time and VO_2peak_, from childhood to young adulthood in an ostensibly healthy Norwegian sample.

## 2 Methods

### 2.1 Study sample

This cohort is recruited from the longitudinal arm of the Physical Activity among Norwegian Children Studies (PANCS) in Norway, where 731 boys and girls participated at both age 9 years (2005–2006) and age 15 years (2011–2012). This longitudinal cohort was distributed across 40 schools, randomly selected by Statistics Norway, covering the different geographical regions of Norway. In 2019, all participants still living in Norway were deemed eligible for inclusion in the PANCS follow-up study at age 24 years. As most of the cardiometabolic risk factors were not measured at age 15 years, only the waves at age 9 and 24 years were included in the current paper.

### 2.2 Data collection

#### 2.2.1 Cardiometabolic risk factors

*Body mass index (BMI)* was calculated using the standard formula kg/m^2^. Weight and height were measured to the nearest 0.1 kg (Seca 770 and 877, SECA GmbH, Hamburg, Germany) and 0.1 cm (wall-mounted measuring tape), respectively. At age 9 years, participants wore underwear during the anthropometric measurements whereas, at age 24 years, participants wore light clothing (gym shorts/pants and T-shirt). Thus, we subtracted 0.3 kg from the bodyweight measures at age 24 years to account for clothing.

*Waist circumference* was measured using a tape measure positioned midway between the hip and rib cage, with the measurement taken immediately after exhalation to the nearest 0.1 cm.

*Peak oxygen uptake* (VO_2peak_) was assessed at age 9 years (2005–2006) through a maximum exercise test on an electronically braked cycle ergometer (Ergomedic 839E, Monark, Varberg, Sweden). Oxygen uptake (VO_2_) was measured every 10 s during the last minutes of the test using a portable MetaMax III X oxygen analyser (Cortex Biophysics, Leipzig, Germany), and the mean of the three highest consecutive measurements was used as VO_2peak_. At age 24 years, VO_2peak_ was measured using a modified Balke protocol on treadmill (Woodway, Weil am Rhein, Germany). Oxygen uptake was registered continuously until exhaustion (Oxycon Pro, Jaeger, Würtsburg, Germany), and the highest value across 30 s samples was used. VO_2peak_ at age 9 and 24 years are presented in both absolute terms (L/min) and relative to body weight (ml/kg/min). The protocols used have been described in detail elsewhere [17,18].

*Sedentary time* was measured using hip-worn ActiGraph accelerometers (ActiGraph, Pensacola, FL, USA). ActiGraph model CSA7164 was used at age 9 years and models GT3X+ and GT3X+BT were used at age 24 years. Participants were instructed to wear the monitor during all waking hours – only removing the device for sleep and water-based activities – for a full week at age 24 years, and for 4 consecutive days (including 2 weekend days) at age 9 years due to limited monitor storage capacity of the CSA7164. The accelerometer data were summarised in 10 s epochs, excluding non-wear periods (defined as consecutive spells of zero counts lasting ≥60 min) [19]. A valid day was defined as ≥480 min of wear time, and time spent ≤100 counts per minute was classified as sedentary behaviour [20]. All pre- and post-processing of raw accelerometer data were done in ActiLife v6.13.4.

*Blood pressure* was measured at age 9 years with an Omega™ automatic monitor (Invivo Research, Inc., Orlando, Fl., USA), and with an Omron M2 automatic monitor at age 24 years (Omron Healthcare, Inc., Kyoto, Japan). The blood pressure was measured sitting upright in a relaxed position following a period of at least five minutes rest, with the cuff firmly fastened on the upper left arm. A total of five measurements were made at age 9 years, using the mean of the last three measurements. At age 24 years, a total of three measurements were made where the mean of the last two measurements was used.

Fasting intravenous blood samples were drawn from the antecubital vein of participants aged 9 and 24 years by trained personnel. The samples were then analysed for *LDL-cholesterol, insulin*, and *high-sensitivity C-reactive protein (HS-CRP)*. LDL-cholesterol and HS-CRP were measured using routine enzymatic colorimetric assays, while insulin was measured using fluoroimmunoassay at age 9 years and chemiluminescent immunoassay at age 24 years. All analyses were conducted by qualified laboratory personnel at accredited laboratories.

*Clustered risk (Z-score)* was calculated by standardising (mean 0, standard deviation 1) values of waist circumference, VO_2peak_ inversed (ml/kg/min), systolic blood pressure, LDL-cholesterol, insulin, and HS-CRP and then taking the mean of these values to create a singular cardiometabolic risk Z-score. Standardisation involved first centering the variables by subtracting the variable mean from each value, before scaling to a mean of 0 and standard deviation of 1 by dividing the centered variables by their standard deviation. Higher values indicate an unfavourable cardiometabolic profile, while lower values indicate a favourable cardiometabolic profile [21].

### 2.3 Covariates

Statistics Norway provided information about *parental income* and *parent’s country of birth* in 2011–2012, when the participants were 15 years of age. Total income of both parents was used as a proximal measure of socioeconomic status, where three income groups were created: low (total parental income <750,000 NOK), middle (total parental income 750,000–1,499,999 NOK), and high (total parental income ≥1,500,000 NOK). National background of the participants was categorised as number of parents born in Norway (none, one, two).

*Birthweigh*t and *childhood chronic disorders or medical issues* (e.g., asthma and allergies) were parentally reported when the participants were 9 years of age.

### 2.4 Statistical analysis

All analyses were performed in R v4.3.0 [22]. A visual examination of the associations between the baseline and follow-up measure of the cardiometabolic risk factors revealed no signs of non-linearity. To preserve collected data, multiple imputation was performed using random forest models with predicted mean matching through the *missRanger* R library [23]. This procedure imputed one variable at a time, including all others but anonymous identification variables as predictors [24], creating 100 different complete data sets on all eligible participants at age 24 years (*n* = 708). Analyses were independently performed on each complete dataset, and results were pooled with the *mice* R library using Rubin’s rules to derive a combined point estimate and standard error across all imputations [25,26].

The tracking analyses were performed separately for biological sex, hereon referred to as males and females. Stability coefficients were calculated with linear regression and modelled as the initial measurement of the cardiometabolic risk factors regressed on the follow-up measurement of the same variable. The tracking variables were standardised (mean 0, standard deviation 1), and the stability coefficients could thus take on values between −1 and 1 and be interpreted as longitudinal correlation coefficients. Absolute stability coefficients <0.3 were considered weak tracking, 0.3–0.6 considered moderate tracking, and >0.6 considered strong tracking [27]. Furthermore, we examined whether belonging to the least favourable quartile of the cardiometabolic risk factors at age 9 years was predictive of belonging to the least favourable quartile at age 24 years. The least favourable quartiles were defined as the lowest quartile for VO_2peak_, and the highest quartile for all other risk factors. Odds ratios were computed using logistic regression, comparing participants belonging to the least favourable quartile to peers belonging to any of the other three quartiles. In all models, adjustments were made for baseline age, length of follow-up, baseline height, birthweight, childhood disorders, number of Norwegian parents, parental income, and baseline waist circumference (non-adiposity risk factors only). Crude models were included to display any effect of the adjustments. To account for potential sources of measurement error of the accelerometers, predicted values of sedentary time were used which were extracted from a linear regression model with sedentary time regressed on mean wear time of the accelerometer, a weekday to weekend wear ratio (number of valid weekdays divided by number of valid weekend days), and which month they wore the device. We further included an indication of whether the tracking estimate would be statistically significant at the conventional significance level of 0.05 after controlling for multiple testing [28].

## 3 Results

**Fig. 1:**
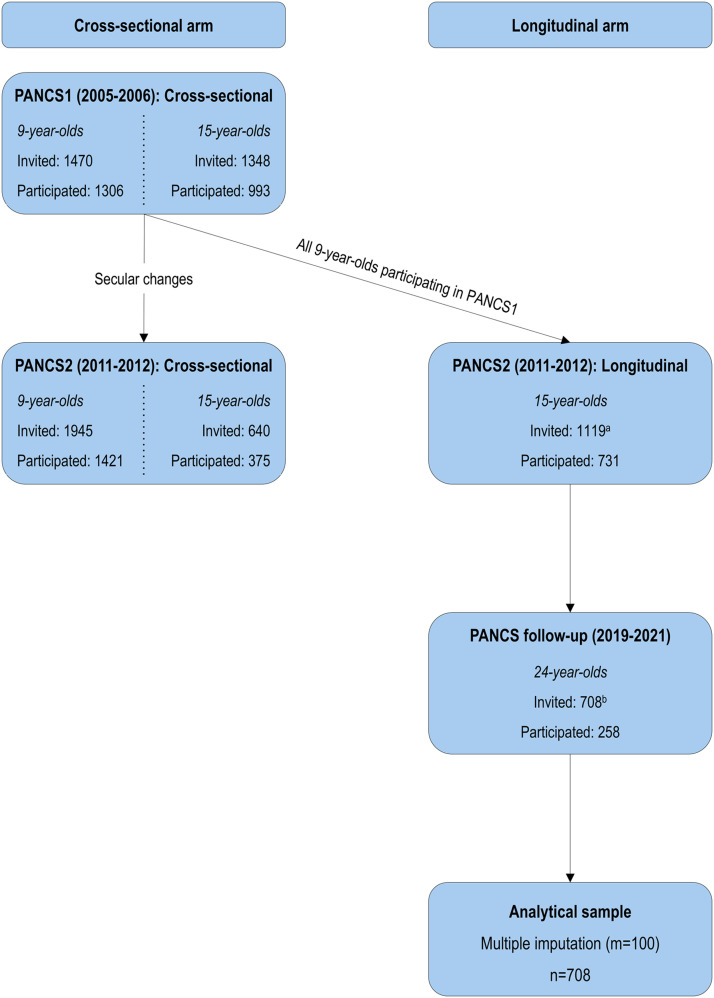
Flow chart of the inclusion process in the cross-sectional and longitudinal study arms of PANCS. ^a^1119 of the 1306 9-years-olds who participated in PANCS1 were identified and invited to participate again in PANCS2 as 15-year-olds. ^b^708 out of 731 participants were still registered in Norway and eligible for PANCS follow-up.

In total, 731 males and females participated at age 9 years. Of these, 708 were still living in Norway and deemed eligible for the PANCS follow-up study at age 24 years (2019–2021), where 258 young adults participated. The main reason for non-participation at age 9 years was a lack of response to the written informed consent form sent to the primary guardians. At age 24 years, the main reasons for non-participation were a lack of interest and scepticism towards domestic traveling during the COVID-19 pandemic. A flow chart of the inclusion process is shown in Fig. 1.

Descriptive statistics are shown in Table 1. In the total sample, the mean age at baseline was 9.6 (SD: 0.4) years and 24.6 (SD: 0.8) years at follow-up – giving an average length of follow-up of 15 (SD: 0.7) years. Anthropometric variables and cardiometabolic risk factors were mostly similar between sexes at age 9 years. Males had significantly higher mean levels of height, weight, waist circumference, sedentary time, VO_2peak_, and systolic blood pressure as compared to the females at age 24. Females comprised 48 % of the total sample at age 9 years, and 55 % at age 24 years. The proportion of males and females belonging to the different categorical levels of parental income, number of Norwegian parents, birthweight, and occurrence of any childhood disorder was comparable at both age 9 and 24 years.

**Table 1:** Descriptive statistics for females and males at 9 and 24 years of age.

		PANCS1 (2005–2006)		PANCS follow-up (2019–2021)	
Variable	Missing	Female n = 340^a^	Male n = 368^a^	Female n = 141^a^	Male n = 117^a^
Age (yrs)	32 %	9.6 (0.4)	9.6 (0.4)	24.5 (0.8)	24.7 (0.7)
Height (cm)	32 %	137.9 (6.3)	139.8 (6.1)	167.5 (5.9)	182.9 (7.1)
Weight (kg)	32 %	33.0 (6.7)	33.7 (6.1)	67.6 (13.6)	79.6 (12.3)
Parental Income (2011–2012)	0.1 %				
Low		71 (21 %)	72 (20 %)	26 (18 %)	18 (15 %)
Middle		197 (58 %)	221 (60 %)	85 (61 %)	68 (59 %)
High		71 (21 %)	74 (20 %)	30 (21 %)	31 (26 %)
Norwegian Parents	0 %				
None		30 (9 %)	34 (9 %)	7 (5 %)	10 (8 %)
One		58 (17 %)	74 (20 %)	21 (15 %)	22 (19 %)
Two		252 (74 %)	260 (71 %)	113 (80 %)	85 (73 %)
Birthweight	12 %				
Low		16 (5 %)	16 (5 %)	7 (5 %)	10 (9 %)
Normal		235 (78 %)	225 (71 %)	104 (79 %)	74 (67 %)
High		52 (17 %)	78 (24 %)	21 (16 %)	27 (24 %)
Childhood disorder	9 %				
No		281 (89 %)	266 (80 %)	122 (90 %)	92 (82 %)
Yes		33 (11 %)	66 (20 %)	13 (10 %)	20 (18 %)
Outcomes					
BMI (kg/m^2^)	32 %	17.3 (2.6)	17.1 (2.3)	24.1 (4.7)	23.8 (3.1)
Waist Circumference (cm)	33 %	62.2 (7.1)	61.6 (7.0)	76.7 (10.8)	84.2 (8.2)
Sedentary time (min/day)	39 %	440 (68)	431 (74)	585 (73)	612 (98)
VO_2peak_ (L/min)	34 %	1.42 (0.18)	1.61 (0.19)	2.81 (0.46)	4.32 (0.72)
VO_2peak_ (ml/kg/min)	34 %	44.1 (6.6)	48.7 (7.3)	42.6 (6.5)	55.3 (8.6)
SBP (mmHg)	33 %	102.6 (7.7)	103.7 (7.6)	125.5 (9.9)	135.8 (12.0)
LDL-cholesterol (mmol/L)	42 %	2.5 (0.7)	2.3 (0.6)	2.9 (0.8)	2.9 (0.8)
Insulin (pmol/L)	52 %	27 (19, 39)	24 (16, 38)	56 (44, 70)	48 (34, 58)
HS-CRP (mg/L)	42 %	0.3 (0.2, 0.6)	0.2 (0.1, 0.5)	1.0 (0.5, 2.3)	0.4 (0.3, 0.7)

BMI: Body mass index; VO_2peak_: Peak oxygen uptake; SBP: Systolic blood pressure; LDL: Low density lipoprotein; HS-CRP: High sensitivity C-reactive protein.

a Mean (SD); Median (IQR); n ( %).

Tracking of cardiometabolic risk factors from 9 to 24 years among females can be found in Table 2. The highest stability coefficients were observed for BMI (0.71, 95 % CI: 0.62, 0.80) and waist circumference (0.67, 95 % CI: 0.57, 0.76), which exhibited strong tracking. Moderate tracking was found for LDL-cholesterol (0.53, 95 % CI: 0.41, 0.65), clustered risk Z-score (0.48, 95 % CI: 0.37, 0.60), relative VO_2peak_ (0.40, 95 % CI: 0.23, 0.56), and absolute VO_2peak_ (0.35, 95 % CI: 0.21, 0.50). Systolic blood pressure (0.29, 95 % CI: 0.16, 0.42), fasting insulin (0.07, 95 % CI: −0.08, 0.21), HS-CRP (0.07, 95 % CI: −0.08, 0.22), and sedentary time (0.06, 95 % CI: −0.08, 0.20) demonstrated weak to no tracking from age 9 to 24 years. Generally, for the cardiometabolic risk factors exhibiting moderate to strong tracking, the females in the least favourable quartiles at age 9 years were substantially more likely to be in these quartiles at age 24 years than peers belonging to any other quartile at baseline. For example, females in the highest quartile of the clustered risk Z-score at age 9 years were almost 6 times more likely to be in the highest quartile at age 24 years compared to females belonging to any of the three lower quartiles at baseline (OR: 5.9, 95 % CI: 2.5, 13.7).

**Table 2:** Tracking of cardiometabolic risk factors among females between the ages of 9 and 24 years (n = 340).

	Adjusted models				Crude models			
	Stability coefficients		Odds ratios^a^		Stability coefficients		Odds ratios^a^	
Outcomes	β (95 % CI)	p-value	OR (95 % CI)	p-value	β (95 % CI)	p-value	OR (95 % CI)	p-value
BMI (kg/m^2^)	0.71 (0.62, 0.80)	<0.001*	17.0 (7.7, 37.6)	<0.001*	0.74 (0.66, 0.83)	<0.001*	16.0 (8.0, 32.1)	<0.001*
Waist circumference (cm)	0.67 (0.57, 0.76)	<0.001*	12.9 (5.7, 29.1)	<0.001*	0.73 (0.65, 0.81)	<0.001*	16.2 (7.6, 34.1)	<0.001*
Sedentary time (min/day)	0.06 (−0.08, 0.20)	0.374	1.14 (0.53, 2.44)	0.730	0.07 (−0.07, 0.20)	0.324	1.21 (0.59, 2.49)	0.597
VO_2peak_ (L/min)	0.35 (0.21, 0.50)	<0.001*	1.99 (0.91, 4.36)	0.087	0.52 (0.41, 0.65)	<0.001*	4.0 (2.1, 7.5)	<0.001*
VO_2peak_ (ml/kg/min)	0.40 (0.23, 0.56)	<0.001*	1.95 (0.78, 4.93)	0.155	0.55 (0.45, 0.65)	<0.001*	6.0 (3.2, 11.4)	<0.001*
SBP (mmHg)	0.29 (0.16, 0.42)	<0.001*	2.6 (1.3, 5.6)	0.010*	0.33 (0.20, 0.46)	<0.001*	3.0 (1.5, 6.0)	0.002*
LDL-cholesterol (mmol/L)	0.53 (0.41, 0.65)	<0.001*	5.7 (2.6, 12.7)	<0.001*	0.53 (0.41, 0.66)	<0.001*	4.6 (2.3, 9.3)	<0.001*
Insulin (pmol/L)	0.07 (−0.08, 0.21)	0.358	1.03 (0.45, 2.34)	0.942	0.25 (0.11, 0.39)	<0.001*	1.94 (1.01, 3.76)	0.048
HS-CRP (mg/L)	0.07 (−0.08, 0.22)	0.330	1.04 (0.42, 2.61)	0.929	0.17 (0.02, 0.32)	0.030	1.70 (0.79, 3.69)	0.175
Clustered Z-score	0.48 (0.37, 0.60)	<0.001*	5.9 (2.5, 13.7)	<0.001*	0.50 (0.40, 0.61)	<0.001*	6.8 (3.1, 14.9)	<0.001*

BMI: Body mass index; VO_2peak_: Peak oxygen uptake; SBP: Systolic blood pressure; LDL: Low density lipoprotein; HS-CRP: High sensitivity C-reactive protein.

a Odds of being in the least favourable quartile in young adulthood if belonging to the least favourable quartile in childhood (lowest quartile for VO_2peak_, highest quartile for all other variables). The reference group is participants belonging to any of the three other quartiles in childhood.

⁎ Statistically significant after correction for multiple comparisons.

The corresponding tracking analysis among males is displayed in Table 3. BMI tracked strongly from age 9 to 24 years with a stability coefficient of 0.69 (95 % CI: 0.59, 0.79). Waist circumference (0.59, 95 % CI: 0.50, 0.68), relative VO_2peak_ (0.57, 95 % CI: 0.42, 0.72), LDL-cholesterol (0.45, 95 % CI: 0.33, 0.58), clustered risk Z-score (0.50, 95 % CI: 0.38, 0.62), absolute VO_2peak_ (0.45, 95 % CI: 0.42, 0.72), and systolic blood pressure (0.31, 95 % CI: 0.19, 0.44) all demonstrated moderate tracking. Weak to no tracking was found for insulin (0.14, 95 % CI: 0.00, 0.27), sedentary time (0.09, 95 % CI: −0.04, 0.22), and HS-CRP (0.03, 95 % CI: −0.10, 0.17). Like the females, being in the least favourable quartile at age 9 years was highly predictive of status at age 24 years for the cardiometabolic risk factors demonstrating moderate to strong tracking. Males in the least favourable quartile of the clustered risk Z-score at age 9 years were over five times more likely to be in the least favourable quartile at age 24 years as compared to peers belonging to any other quartile at age 9 years (OR: 5.4, 95 % CI: 2.5, 11.4).

**Table 3:** Tracking of cardiometabolic risk factors among males between the ages of 9 and 24 years (n = 368).

	Adjusted models				Crude models			
	Stability coefficients		Odds ratios^a^		Stability coefficients		Odds ratios^a^	
Outcomes	β (95 % CI)	p-value	OR (95 % CI)	p-value	β (95 % CI)	p-value	OR (95 % CI)	p-value
BMI (kg/m^2^)	0.69 (0.59, 0.78)	<0.001*	10.4 (5.0, 21.6)	<0.001*	0.74 (0.66, 0.82)	<0.001*	12.7 (6.5, 24.9)	<0.001*
Waist circumference (cm)	0.59 (0.50, 0.68)	<0.001*	6.6 (3.1, 14.0)	<0.001*	0.69 (0.61, 0.78)	<0.001*	9.1 (4.7, 17.5)	<0.001*
Sedentary time (min/day)	0.09 (−0.04, 0.22)	0.174	1.05 (0.47, 2.39)	0.899	0.07 (−0.06, 0.20)	0.317	0.98 (0.46, 2.11)	0.964
VO_2peak_ (L/min)	0.46 (0.35, 0.57)	<0.001*	5.3 (2.2, 12.9)	<0.001*	0.62 (0.53, 0.71)	<0.001*	7.9 (3.9, 15.9)	<0.001*
VO_2peak_ (ml/kg/min)	0.57 (0.42, 0.72)	<0.001*	4.7 (2.0, 11.2)	<0.001*	0.57 (0.47, 0.66)	<0.001*	6.9 (3.6, 13.0)	<0.001*
SBP (mmHg)	0.31 (0.19, 0.44)	<0.001*	2.7 (1.3, 5.4)	0.005*	0.36 (0.24, 0.48)	<0.001*	2.9 (1.5, 5.6)	0.001*
LDL-cholesterol (mmol/L)	0.45 (0.33, 0.58)	<0.001*	6.2 (2.7, 14.1)	<0.001*	0.49 (0.36, 0.61)	<0.001*	6.0 (2.9, 12.5)	<0.001*
Insulin (pmol/L)	0.14 (0.00, 0.27)	0.045	1.89 (0.89, 4.03)	0.098	0.28 (0.15, 0.41)	<0.001*	2.6 (1.4, 5.1)	0.004*
HS-CRP (mg/L)	0.03 (−0.10, 0.17)	0.660	1.43 (0.64, 3.18)	0.376	0.09 (−0.05, 0.23)	0.210	1.90 (0.91, 3.95)	0.085
Clustered Z-score	0.50 (0.38, 0.62)	<0.001*	5.4 (2.5, 11.4)	<0.001*	0.54 (0.43, 0.65)	<0.001*	6.2 (3.2, 12.2)	<0.001*

BMI: Body mass index; VO_2peak_: Peak oxygen uptake; SBP: Systolic blood pressure; LDL: Low density lipoprotein; HS-CRP: High sensitivity C-reactive protein.

a Odds of being in the least favourable quartile in young adulthood if belonging to the least favourable quartile in childhood (lowest quartile for VO_2peak_, highest quartile for all other variables). The reference group is participants belonging to any of the three other quartiles in childhood.

⁎ Statistically significant after correction for multiple comparisons.

The majority of the cardiometabolic risk factors tracked similarly between males and females. However, waist circumference tracked somewhat stronger among females while VO_2peak_ tracked stronger among males.

As a sensitivity analysis, a complete-case analysis was conducted (Supplementary file, Table S1 & Table S2). In general, the stability coefficients were comparable to those of the multiply imputed data set, apart for somewhat higher tracking of systolic blood pressure and LDL-cholesterol in the complete-case analysis. The odds ratios, however, were generally smaller in magnitude using the complete cases, which could be due to the large amount of both observed and missing data being omitted from the models through listwise deletion (mean observations among females: 111; mean observations among males: 94).

## 4 Discussion

Several cardiometabolic risk factors demonstrated moderate to strong tracking from childhood to young adulthood, with BMI and waist circumference being the most prominent, followed by VO_2peak_, LDL-cholesterol, and the clustered cardiometabolic risk score. Systolic blood pressure demonstrated moderate tracking, while other risk factors did not track. Furthermore, belonging to the least favourable quartile of the cardiometabolic risk factors exhibiting moderate to strong tracking at age 9 years was highly predictive of risk status at age 24 years. The tracking magnitude was comparable between males and females except for waist circumference and VO_2peak_.

The observed moderate to strong tracking of adiposity and blood lipids aligns with findings from various studies spanning childhood to young adulthood [10,[29], [30], [31], [32], [33]]. Similarly, the tracking of systolic blood pressure is consistent with previous research, although some studies report somewhat stronger tracking [10,15,34,35]. However, the systolic blood pressure measurements at age 24 in the current study showed substantial intra-person variation, averaging approximately 10 mmHg between the lowest and highest values of the three measurements conducted. This variability might have introduced undesired noise, potentially distorting the tracking analysis. Furthermore, both females and males demonstrated no or weak tracking for insulin level and HS-CRP. The low stability of these markers in our study may be attributed to the biological variation associated with both insulin and HS-CRP [36,37]. Indeed, an inherent challenge to tracking analyses is that tracking cannot be higher than the reliability of the measurement method [38], which may lead to underestimating tracking magnitude of fluctuating cardiometabolic risk factors. Furthermore, the stability of various cardiometabolic risk factors in young people may be influenced by both age at initial measurement and length of follow-up [39,40], implying that several markers of cardiometabolic risk may vary throughout young age due to developmental factors such as hormonal and maturational changes [14]. However, the clustered cardiometabolic risk score exhibited robust tracking from age 9 to 24 years, supporting the limited number of previous studies [14], although variations in assessment methods and definitions of clustered cardiometabolic risk exist.

Our measurements of direct VO_2peak_ by indirect calorimetry and device-measured sedentary time in both childhood and young adulthood are novel. We observed distinct tracking patterns for these two risk factors. VO_2peak_ exhibited moderate to strong tracking, especially among males, whereas sedentary time showed no tracking. While several studies have explored tracking of cardiorespiratory fitness from childhood to adulthood [41], few have used directly measured VO_2peak_. Previous investigations, such as the European Youth Heart Study, the Amsterdam Growth and Health Study, and the Danish Youth and Sport Study, have also examined tracking of directly measured VO_2peak_ but with different age at baseline and length of follow-up [10,42,43]. Our findings mostly corroborate these earlier observations. Interestingly, Andersen et al. observed the strongest tracking among females [42], contrasting with our findings. Earlier research indicates that relative VO_2peak_ (ml/kg/min) remains reasonably stable during puberty among males but gradually decline among females [44]. This, along with different age at baseline, may partly explain the sex differences.

We know of no other studies examining the tracking of device-based sedentary time from childhood to young adulthood. A recent paper based on data from the International Children’s Accelerometery Database (ICAD) found moderate tracking of total sedentary time in young people, where the mean baseline age was 10.3 years and length of follow-up 2.7 years [45]. However, a systematic review by Biddle and colleagues noted varying stability of sedentary behaviour in youth [46]. A noteworthy implication of our findings is that sedentary time may exhibit a lower degree of tracking from childhood to young adulthood, as compared to cardiometabolic risk factors such as BMI and VO_2peak_ – which both have strong heritable components [47,48]. Particularly measures of adiposity in the current paper – BMI and waist circumference – showed strong tracking, and prior studies emphasise the challenges of weight loss and long-term weight loss maintenance [49,50]. The contrasting weak tracking of sedentary time found in the current paper may suggest that sedentary behaviour could be more modifiable than more stable cardiometabolic risk factors such as BMI and waist circumference, highlighting the potential for interventions targeting a reduction in sedentary time and increase in time spent in moderate-to-vigorous physical activity. Simply reducing or breaking up sedentary behaviour may be beneficial for cardiometabolic risk [51], and shifting sedentary time to physical activity of at least moderate intensity among sedentary adults have shown to improve cardiometabolic risk factors such as blood lipid profile and cardiorespiratory fitness [52].

There are some limitations to this study. Most measured variables remained consistent in terms of methods used in childhood and young adulthood, with a few exceptions. Notably, different ActiGraph accelerometer models were used at age 9 and 24 years to measure sedentary time, and the ergometer used for maximal exercise testing of VO_2peak_ shifted from a stationary bicycle in childhood to a treadmill in young adulthood. While the absolute time spent sedentary and the VO_2peak_ measurements may differ in youth depending on these measurement methods [53,54], results from both the two accelerometer models and the two ergometers are highly correlated [55,56] – thus minimising the threat to the validity of our tracking analyses. Another limitation stems from the loss to follow-up in young adulthood. Multiple imputation was used to address missing data, assuming that data was missing at random (MAR) and thus depending on observed data [57]. A comparison of participants who participated at both age 9 and 24 years with those who dropped out revealed some differences in BMI and waist circumference, male-to-female ratio, parental income, national background, and occurrence of childhood disorder (Supplementary file, Table S3). While the MAR assumption cannot be directly verified, our extensive dataset with numerous variables increases the likelihood of a MAR mechanism behind the missing data [58]. Multiple imputation is often favoured over complete-case analysis as it provides valid inferences under the MAR assumption whereas listwise deletion could potentially introduce bias [59], especially when fully-observed units are scarce [60]. A quality check of the multiple imputation process, detailed in the Supplementary file (Figure S1), confirms that the imputation avoided generating implausible values and maintained the observed distribution of variables.

In conclusion, this study provides novel information about the tracking of device-based sedentary time, directly measured VO_2peak_, and a clustered cardiometabolic risk score from childhood to young adulthood. The most prominent tracking among the cardiometabolic risk factors were found for BMI, waist circumference, VO_2peak_, LDL-cholesterol, and the clustered cardiometabolic risk score – showing small differences between sexes. These findings underscore the importance of early intervention, as an unfavourable cardiometabolic profile in childhood is likely to endure into young adulthood. Sedentary time demonstrated a lower degree of tracking than several other established risk factors and may be more modifiable and thus a promising target for intervention.

## Data Availability Statement

De-identified individual participant data that underlie the reported results can be accessed upon reasonable request after publication. Requests should be directed to the corresponding author and needs to be approved by the principal investigators (UE and SAA). Analytical code (R scripts) will be made available upon request.

## Source of funding

The Physical Activity among Norwegian Children Study (PANCS) 1 & 2 were funded by the Norwegian Directorate of Health. The PANCS follow-up study was funded by the Research Council of Norway (#249932) and the Norwegian School of Sport Sciences. The funders had no role in the study design, conduct of the study, data collection, data analysis and interpretation, or in writing and publishing the manuscript.

## Ethical review statement

Ethical approval was obtained from The Regional Committee for Medical Research Ethics (PANCS1, REK Sør: S-04305) and the Internal Ethics Committee at the Norwegian School of Sports Sciences (PANCS follow-up, ref. number 73-300818). The Norwegian Social Science Data Services approved the processing of personal data in all three studies (ref. numbers 12166, 25870 and 276580). Written informed consent was obtained from all participants, and their primary guardians (PANCS1 & 2). All studies were conducted in accordance with the ethical standards of the 1964 Helsinki Declaration and its later amendments.

## CRediT authorship contribution statement

**Anders Husøy:** Writing – original draft, Project administration, Formal analysis, Data curation. **Elin Kolle:** Writing – review & editing. **Jostein Steene-Johannessen:** Writing – review & editing. **Lars Bo Andersen:** Writing – review & editing, Conceptualization. **Ulf Ekelund:** Writing – review & editing, Funding acquisition. **Sigmund Alfred Anderssen:** Writing – review & editing, Conceptualization.

## Declaration of competing interest

The authors declare the following financial interests/personal relationships which may be considered as potential competing interests:

Ulf Ekelund reports financial support was provided by Research Council of Norway. If there are other authors, they declare that they have no known competing financial interests or personal relationships that could have appeared to influence the work reported in this paper.

## Appendix Supplementary materials

Image, application 1
