# Supplementary material for: Tracking of device-measured sedentary time, cardiorespiratory fitness, and cardiometabolic risk factors from childhood to young adulthood
Source: Am J Prev Cardiol. 2025 Sep 22;24:101313. doi: 10.1016/j.ajpc.2025.101313 (PMC12549386; doi:10.1016/j.ajpc.2025.101313)
Supplement: Supplementary file 1 [file mmc1.docx]

**Supplementary material for:**

Tracking of device-measured sedentary time, cardiorespiratory fitness, and cardiometabolic risk factors from childhood to young adulthood

Husøy, A.0F0F0F0F3F^[[1]](#footnote-2),*^, Kolle, E.^1^, Steene-Johannessen, J.^1^, Andersen, L.B.1F1F1F1F4F^[[2]](#footnote-3)^, Ekelund, U.^1,^2F2F2F2F5F^[[3]](#footnote-4)^, Anderssen, S.A.^1^

***Corresponding author:** Anders Husøy **(**[andershu@nih.no](mailto:andershu@nih.no))

[Complete-case analysis (listwise deletion) 2](#_Toc187396266)

[Comparison of observed and imputed values 4](#_Toc187396267)

[Comparison of completers and non-completers 5](#_Toc187396268)

# Complete-case analysis (listwise deletion)

**Table S1.** Sensitivity analysis of tracking among females between the ages of 9 and 24 years (complete-case analysis; n_mean_=111)).

|  |  | | Adjusted models | | | | |  | | |  | |  | | Crude models | | | | |  | | |  |  |
| --- | --- | --- | --- | --- | --- | --- | --- | --- | --- | --- | --- | --- | --- | --- | --- | --- | --- | --- | --- | --- | --- | --- | --- | --- |
|  | **Stability coefficients** | | | | | **Odds ratios^a^** | | | | |  | | **Stability coefficients** | | | | | **Odds ratios^a^** | | | | | |  |
| *Outcomes* | *β (95% CI)* | | | *p-value* | | *OR (95% CI)* | | | *p-value* | |  | | *β (95% CI)* | | | *p-value* | | *OR (95% CI)* | | | *p-value* | | |  |
| BMI (kg/m^2^) | 0.82 (0.65, 1.00) | | | <0.001 | | 8.6 (3.0, 27.3) | | | <0.001 | |  | | 0.79 (0.64, 0.95) | | | <0.001 | | 7.3 (3.0, 18.4) | | | <0.001 | | |  |
| Waist circumference (cm) | 0.67 (0.48, 0.86) | | | <0.001 | | 5.4 (1.9, 16.7) | | | 0.002 | |  | | 0.69 (0.53, 0.85) | | | <0.001 | | 8.9 (3.6, 23.3) | | | <0.001 | | |  |
| Sedentary time (min/day) | −0.01 (−0.30, 0.28) | | | 0.951 | | 0.45 (0.11, 1.55) | | | 0.232 | |  | | 0.05 (−0.18, 0.28) | | | 0.678 | | 1.04 (0.37, 2.67) | | | 0.944 | | |  |
| VO_2peak_ (L/min) | | 0.40 (0.15, 0.66) | | | 0.002 | | 1.58 (0.49, 5.01) | | | 0.436 | |  | | 0.41 (0.23, 0.59) | | | <0.001 | | 2.7 (1.1, 6.2) | | | 0.023 | | |
| VO_2peak_ (ml/kg/min) | 0.21 (−0.08, 0.51) | | | 0.154 | | 0.48 (0.11, 1.94) | | | 0.318 | |  | | 0.39 (0.21, 0.57) | | | <0.001 | | 1.61 (0.62, 3.97) | | | 0.312 | | |  |
| SBP (mmHg) | 0.40 (0.22, 0.57) | | | <0.001 | | 4.0 (1.4, 11.6) | | | 0.010 | |  | | 0.45 (0.28, 0.61) | | | <0.001 | | 4.1 (1.7, 10.0) | | | 0.002 | | |  |
| LDL-cholesterol (mmol/L) | 0.69 (0.52, 0.86) | | | <0.001 | | 6.5 (2.1, 22.4) | | | 0.002 | |  | | 0.68 (0.52, 0.84) | | | <0.001 | | 4.5 (1.8, 11.9) | | | 0.002 | | |  |
| Insulin (pmol/L) | 0.05 (−0.24, 0.35) | | | 0.709 | | 0.61 (0.09, 3.27) | | | 0.577 | |  | | 0.16 (−0.08, 0.40) | | | 0.194 | | 1.14 (0.31, 3.87) | | | 0.833 | | |  |
| HS-CRP (mg/L) | 0.17 (−0.07, 0.42) | | | 0.153 | | 0.54 (0.12, 2.04) | | | 0.383 | |  | | 0.26 (0.06, 0.45) | | | 0.008 | | 0.87 (0.26, 2.53) | | | 0.804 | | |  |
| BMI: Body mass index; VO_2peak_: Peak oxygen uptake; SBP: Systolic blood pressure; LDL: Low density lipoprotein; HS-CRP: High sensitivity C-reactive protein.  ^a^ Odds of being in the least favourable quartile in young adulthood if belonging to the least favourable quartile in childhood (lowest quartile for VO_2peak_, highest quartile for all other variables). The reference group is participants belonging to any of the three other quartiles in childhood. | | | | | | | | | | | | | | | | | | | | | | | | |

**Table S2.** Sensitivity analysis of tracking among males between the ages of 9 and 24 years (complete-case analysis; n_mean_=94).

|  |  | | Adjusted models | | | | |  | | |  | |  | | Crude models | | | | |  | | |  |  |
| --- | --- | --- | --- | --- | --- | --- | --- | --- | --- | --- | --- | --- | --- | --- | --- | --- | --- | --- | --- | --- | --- | --- | --- | --- |
|  | **Stability coefficients** | | | | | **Odds ratios^a^** | | | | |  | | **Stability coefficients** | | | | | **Odds ratios^a^** | | | | | |  |
| *Outcomes* | *β (95% CI)* | | | *p-value* | | *OR (95% CI)* | | | *p-value* | |  | | *β (95% CI)* | | | *p-value* | | *OR (95% CI)* | | | *p-value* | | |  |
| BMI (kg/m^2^) | 0.59 (0.43, 0.76) | | | <0.001 | | 3.8 (1.3, 11.0) | | | 0.013 | |  | | 0.67 (0.50, 0.83) | | | <0.001 | | 6.1 (2.4, 15.9) | | | <0.001 | | |  |
| Waist circumference (cm) | 0.52 (0.34, 0.70) | | | <0.001 | | 1.58 (0.48, 5.07) | | | 0.442 | |  | | 0.61 (0.44, 0.78) | | | <0.001 | | 3.9 (1.5, 10.2) | | | 0.005 | | |  |
| Sedentary time (min/day) | 0.07 (−0.21, 0.35) | | | 0.607 | | 0.68 (0.16, 2.59) | | | 0.590 | |  | | 0.19 (−0.04, 0.43) | | | 0.098 | | 0.71 (0.21, 2.05) | | | 0.546 | | |  |
| VO_2peak_ (L/min) | | 0.54 (0.35, 0.73) | | | <0.001 | | 48.8 (7.9, 527.4) | | | <0.001 | |  | | 0.61 (0.46, 0.76) | | | <0.001 | | 13.1 (4.8, 38.9) | | | <0.001 | | |
| VO_2peak_ (ml/kg/min) | 0.62 (0.36, 0.88) | | | <0.001 | | 2.7 (0.6, 12.6) | | | 0.200 | |  | | 0.48 (0.29, 0.67) | | | <0.001 | | 1.98 (0.74, 5.15) | | | 0.163 | | |  |
| SBP (mmHg) | 0.52 (0.36, 0.69) | | | <0.001 | | 5.7 (1.8, 19.3) | | | 0.003 | |  | | 0.47 (0.31, 0.64) | | | <0.001 | | 4.2 (1.7, 10.9) | | | 0.002 | | |  |
| LDL-cholesterol (mmol/L) | 0.55 (0.34, 0.76) | | | <0.001 | | 14.3 (3.7, 68.5) | | | <0.001 | |  | | 0.53 (0.33, 0.74) | | | <0.001 | | 6.7 (2.3, 20.5) | | | <0.001 | | |  |
| Insulin (pmol/L) | 0.19 (−0.19, 0.57) | | | 0.308 | | 1.34 (0.12, 19.46) | | | 0.816 | |  | | 0.13 (−0.20, 0.45) | | | 0.427 | | 1.44 (0.26, 6.94) | | | 0.657 | | |  |
| HS-CRP (mg/L) | 0.00 (−0.23, 0.23) | | | 0.988 | | 0.32 (0.05, 1.56) | | | 0.184 | |  | | −0.05 (−0.26, 0.16) | | | 0.666 | | 0.58 (0.12, 2.01) | | | 0.426 | | |  |
| BMI: Body mass index; VO_2peak_: Peak oxygen uptake; SBP: Systolic blood pressure; LDL: Low density lipoprotein; HS-CRP: High sensitivity C-reactive protein.  ^a^ Odds of being in the least favourable quartile in young adulthood if belonging to the least favourable quartile in childhood (lowest quartile for VO_2max_, highest quartile for all other variables). The reference group is participants belonging to any of the three other quartiles in childhood. | | | | | | | | | | | | | | | | | | | | | | | | |

# Comparison of observed and imputed values


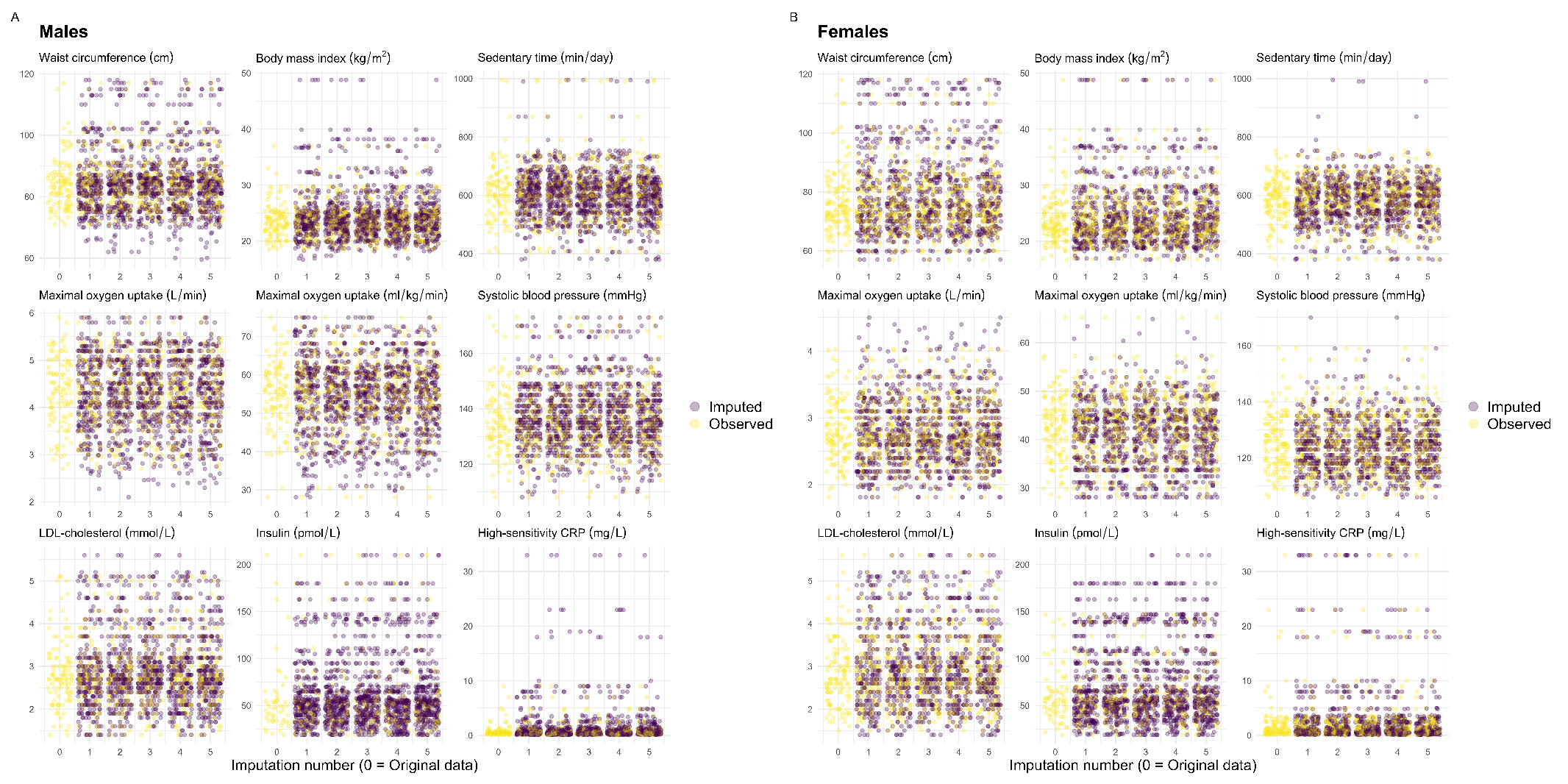


**Figure S1**. Comparison of observed and imputed values of the outcomes at age 24, for the first 5 imputations, among males and females.

# Comparison of completers and non-completers

**Table S3.** Comparison of completers of all three studies with participants who dropped out among all eligible participants for the PANCS follow-up study (n=708).

|  | **Age 9** | | |
| --- | --- | --- | --- |
| *Variable* | *Missing* | *Completer* (n=258)*^a^* | *Dropout* (n=450)*^a^* |
| Age (yrs) | 0% | 9.6 (0.4) | 9.6 (0.4) |
| Height (cm) | 0.8% | 138.7 (6.1) | 139.2 (6.7) |
| Weight (kg) | 0.8% | 32.7 (5.7) | 33.8 (6.9) |
| Birth weight (kg) | 12% | 3.528 (0.648) | 3.545 (0.598) |
| Sex | 0% |  |  |
| Female |  | 141 (55%) | 199 (44%) |
| Male |  | 117 (45%) | 251 (56%) |
| Parental income | 0.1% |  |  |
| Low |  | 44 (17%) | 99 (22%) |
| Middle |  | 153 (59%) | 265 (59%) |
| High |  | 61 (24%) | 85 (19%) |
| Norwegian parents | 0% |  |  |
| None |  | 17 (6.6%) | 47 (10%) |
| One |  | 43 (17%) | 89 (20%) |
| Two |  | 198 (77%) | 314 (70%) |
| Childhood disorder | 8.8% |  |  |
| No |  | 214 (87%) | 333 (83%) |
| Yes |  | 33 (13%) | 66 (17%) |
| *Accelerometer data* |  |  |  |
| Valid wear days | 13% | 3.7 (0.6) | 3.7 (0.6) |
| Wear time (hrs/day) | 13% | 13.3 (1.0) | 13.2 (1.2) |
| *Outcomes* |  |  |  |
| BMI (kg/m^2^) | 1.0% | 16.9 (2.1) | 17.4 (2.6) |
| Waist circumference (cm) | 0.8% | 61.5 (6.2) | 62.1 (7.5) |
| Sedentary time (min/day) | 13% | 439.2 (66.8) | 433.0 (73.7) |
| VO_2peak_ (ml/kg/min) | 2.8% | 46.8 (6.8) | 46.4 (7.6) |
| Systolic blood pressure (mmHg) | 1.4% | 102.7 (7.4) | 103.5 (7.8) |
| LDL-cholesterol (mmol/L) | 17% | 2.4 (0.7) | 2.4 (0.7) |
| Insulin (pmol/L) | 21% | 24.5 (17.0, 37.0) | 27.0 (18.0, 39.3) |
| HS-CRP (mg/L) | 18% | 0.3 (0.1, 0.6) | 0.3 (0.1, 0.6) |
| **BMI: Body Mass Index; VO_2peak_: Peak oxygen uptake; LDL: Low-density lipoprotein; HS-CRP: High-sensitivity C-reactive protein**  *^a^* Mean (SD); n (%); Median (IQR) | | | |
| Note: Completers defined as having participated in all three studies, otherwise dropout. | | | |

1. Department of Sports Medicine, Norwegian School of Sport Sciences, N-0806 Oslo, Norway [↑](#footnote-ref-2)
2. Department of Sport, Food and Natural Sciences, Western Norway University of Applied Sciences, N-6856 Sogndal, Norway [↑](#footnote-ref-3)
3. Department of Chronic Diseases, Norwegian Institute of Public Health, N-0213 Oslo, Norway [↑](#footnote-ref-4)
